# Antibiotic-Induced Change of Bacterial Communities Associated with the Copepod *Nitocra spinipes*

**DOI:** 10.1371/journal.pone.0033107

**Published:** 2012-03-12

**Authors:** Anna Edlund, Karin Ek, Magnus Breitholtz, Elena Gorokhova

**Affiliations:** 1 Department of Systems Ecology, Stockholm University, Stockholm, Sweden; 2 Department of Applied Environmental Science, Stockholm University, Stockholm, Sweden; U. S. Salinity Lab, United States of America

## Abstract

Environmental pressures, such as physical factors, diet and contaminants may affect interactions between microbial symbionts and their multicellular hosts. Despite obvious relevance, effects of antimicrobial contaminants on host-symbiont relations in non-target aquatic organisms are largely unknown. We show that exposure to antibiotics had negative effects on survival and juvenile development of the copepod *Nitocra spinipes* and caused significant alterations in copepod-associated bacterial communities. The significant positive correlations between indices of copepod development and bacterial diversity indicate that disruption of the microflora was likely to be an important factor behind retarded juvenile development in the experimental animals. Moreover, as evidenced by ribotype distribution in the bacterial clone libraries, the exposure to antibiotics caused a shift in dominance from Betaproteobacteria to *Cardinium* bacteria; the latter have been shown to cause reproductive manipulations in various terrestrial arthropods. Thus, in addition to providing evidence that the antibiotic-induced perturbation of the microbial community associates with reductions in fitness-related traits of the host, this study is the first record of a copepod serving as a host for endosymbiotic *Cardinium*. Taken together, our results suggest that (1) antimicrobial substances and possibly other stressors can affect micobiome and symbiont-mediated interactions in copepods and other hosts, and (2) *Cardinium* endosymbionts may occur in other copepods and affect reproduction of their hosts.

## Introduction

Bacteria mediate a variety of interactions within and between organisms. During the past 500 million years they have evolved diverse mechanisms to gain entry and proliferate in multicellular eukaryotes [1], with their effects on hosts ranging from harmful to beneficial [1], [2]. Examples of such effects include reproduction disorders caused by the parasite *Wolbachia* proliferating in arthropod ovaries and testes [3] and beneficial effects from the symbiont *Buchnera* providing essential amino acids to their aphid hosts [4]. The greatest impact of symbiotic bacteria upon hosts can be found in the highly diverse phylum Arthropoda, where obligate host-bacteria associations have been well studied from reproductive and nutrition perspectives, especially in insects [1], [5]. A recently discovered bacterial symbiont causing reproductive disorders in terrestrial arthropods is the *Cardinium* bacteria [6], [7]. Since its discovery, it has been found in four orders of insects and in 6–7% of arthropods, a small number compared to *Wolbachia,* which has been detected in all insects orders and in 66% of arthropods [7]–[9]; however the number of potential *Cardinium* host species tested so far is relatively small. Effects of *Cardinium* bacteria on host fitness are largely unknown, but reproduction disorders in infected populations have been reported for the parasitoid wasp *Encarsia pergandiella* and the spider mites, *Eotetranychnus suginamensis* and *Bryobia sarothamni* [8], [10]–[12].

Whereas the importance of endosymbionts is well appreciated in terrestrial arthropods, virtually nothing is known about their distribution and functioning in copepods, the most abundant aquatic arthropods. In both freshwater and marine environments, these crustaceans are often the key players in pelagic and benthic food webs [13]. They also serve as the main food source for fish larvae in the wild and in aquacultures [14]. In addition, copepods are being increasingly used as test species in ecotoxicology, as a sexually reproducing alternative to the parthenogenic cladoceran *Daphnia* [15]. Of particular interest, therefore, are copepod-bacteria interactions that have a potential to affect growth and reproduction in these animals. Application of advanced microscopy techniques together with molecular and cultivation approaches has shown that diverse copepod-associated bacteria communities exist both inside copepod gut and on exoskeleton [16]–[18]. Therefore, it is highly likely that similar to other arthropods, symbiotic bacteria are important for copepod nutrition, immune responses and reproduction.

Environmental pressures, such as physical factors, diet and contaminants may affect interactions between microbial symbionts and their multicellular hosts. Antibiotics have become environmental contaminants of concern as they are biologically active, which obviously is a part of their nature. In order to be effective, they often have a low biodegradability: 30–90% doses of antibiotics enter the environment in their original form [19]. Antibiotics have been detected in the ng/L to mg/L range in the effluent of sewage treatment plants, surface water and ground water [19]; however, despite obvious relevance, effects of these contaminants on host-symbiont relations in non-target aquatic organisms are largely unknown.

To directly explore associations between bacterial diversity and development of a copepod host as well as effects of antibiotics on these associations, the harpacticoid copepod *Nitocra spinipes* exposed to antibiotics was used as a model system. Three commercially used antibiotics (ciprofloxacin, sulfomethoxazole and trimethoprim) at low concentrations were used to perturb composition and structure of bacterial communities. These antibiotics were selected because they: (1) represent three prominent classes of antibiotics with differing mechanisms of action against bacteria, (2) have been detected in surface waters [19], [20], and (3) have been identified as posing a threat to aquatic environment by risk assessment analysis based on the ratio between predicted environmental concentration and predicted no effect concentration [20]. We hypothesized that this exposure would alter composition of bacterial assemblages associated with the copepods, and that the disrupted copepod-bacteria interactions would cause decreased copepod growth and development. To examine changes in the copepod-associated bacteria following exposure to the antibiotics, we used a 16S rRNA gene clone library approach. To the best of our knowledge, this is the first report on how antibiotics affect both the copepod-associated bacterial community and the development of the host.

## Materials and Methods

### Test organism and study system

*Nitocra spinipes* is a harpacticoid copepod with a worldwide distribution [21]. Animals used in the present study originated from a continuous stock culture maintained in our laboratory; the original strain was isolated from a sediment sample in the Tvären Bay, Baltic Sea, in 1975. The culture is maintained in darkness under semi-static renewal conditions at 22±1°C in 0.03-mm filtered and pasteurized natural brackish water (salinity 6.5‰). Under these conditions, *N. spinipes* has a generation time of about 3 weeks, going through 6 naupliar stages (ca. 5–7 days, body length 0.09 to 0.20 mm) and 5 copepodite stages (ca. 6–10 days, 0.23 to 0.52 mm) before reaching sexual maturity.

### Chemicals, labware and chemical analyses

Three antibiotics were used in the present study; ciprofloxacin (Fluka 17850; purity >98%), trimethoprim (Sigma 92131, purity >99%) and sulfamethoxazole (Fluka 31737, purity 99.9%). All utensils used in this work were either sterile or autoclaved before use. Water samples from each treatment were taken at the termination of the experiment, and antibiotic concentrations were analyzed by a liquid chromatography-tandem mass-spectrometer (LS-MS). Details on the chemical analysis are available in Data S1.

### Experimental set up

To obtain nauplii for all treatments and controls, ∼400 ovigerous females were isolated from the culture one day before the experiment, transferred stepwise to autoclaved synthetic seawater (SS; 6‰; Instant Ocean, Aquarium Systems), and provided with food (alga *Rhodomonas salina*, ∼2.5×10^5^ cells/mL). The females were incubated in darkness for 24 h and hatched nauplii were collected using a wide-mouth pipette.

A single treatment with three replicates for each of the three antibiotics as well as three solvent controls – SS, acetone and DMSO, were used. Stock solution of sulfamethoxazole was prepared in acetone (AnalaR Normapur, 99.97%, BDH Prolabo), trimethoprim in DMSO (max 0.01% H_2_O; purity PA, Scharlau), and ciprofloxacin in SS. The different solvents were used because of solubility differences among the antibiotics. All antibiotics were tested at a sublethal nominal concentration of 5 mg/L, which was selected based on previous ecotoxicity studies with these substances and microcrustaceans [20], [22], [23]. Test solutions were prepared at the start of the experiment and at test medium renewals by diluting controls and stock solutions in SS. The final concentrations of the solvents did not exceed 0.1 µL/L and were the same in both antibiotic exposures and solvent controls. Measured antibiotic concentrations (mean±SD) in the treatments did not deviate substantially from the nominal concentrations: 3.0±0.4 mg/L for ciprofloxacin, 7.7±0.8 mg/L for sulfomethoxazole, and 4.7±0.3 mg/L for trimethoprim.

Newly hatched nauplii were transferred to sterile 6-well microplates (Corning, USA) at 22–23°C, 50 nauplii/well in 5 mL of SS and ∼3.75×10^5^ cells/mL of *R. salina*. Antibiotic exposure started by adding 5 mL of the respective test solution/control water to each test well. Test medium renewal was done twice a week by removing 70% of the media and adding fresh test solution. Fresh algal food suspension was provided to the copepods in the ciprofloxacin and sulfamethoxazole treatments and the corresponding controls on day 11. The total exposure time was 13 days for sulfamethoxazole, ciprofloxacin and the corresponding controls; this period was sufficient for most of the nauplii to develop into copepodites. However, the trimethoprim treatment and the corresponding control were terminated after 11 days due to arrested metamorphosis in the nauplii exposed to the antibiotics and thus increased mortality risk. Therefore, for the downstream analyses (PCR amplification and clone library analyses), we used copepodites for the ciprofloxacin and sulfamethoxazole treatments and nauplii for the trimethoprim treatment. Upon termination of the experiment, the copepods were allowed to empty their guts in SS for 24 hours, transferred to Eppendorf tubes containing 500 µL of molecular grade ethanol, and centrifuged at 2 000× *g* for 3 min. The ethanol was gently discarded and 100 µL of 6% Chelex buffer were added to each tube to proceed with DNA extraction. To achieve an adequate samples size (∼125 individuals), we pooled three experimental replicates within each treatment and control.

### DNA extraction and PCR amplification

DNA was extracted with 10% Chelex [24]. Partial 16S rRNA gene sequences (∼900 bp) were amplified from the extracted DNA using the following degenerate bacterial primers: fD1 (AGAGTTTGATCMTGGCTCAG) [25] and 926r (CCGTCAATTCCTTTRAGTTT) [26]. Each 50 µL PCR contained 35 pmol of each primer, a ready to use PCR master mix (Fermentas, Hanover, MD), 5 µg BSA (Fermentas) and 1 µL of template DNA. Cycling program was performed using a GeneAmp PCR System 2400 thermocycler (Applied Biosystems, Foster City, CA) as follows: 5 min at 95°C followed by 33 cycles of 40 s at 94°C, 1 min at 56°C, 1 min at 72°C and extension at 72°C for 7 min. PCR products were purified and concentrated by using the DNA Clean & Concentrator −5 Kit (Zymo Research, CA) prior to cloning.

### Cloning and sequencing

In total, 6 clone libraries were created from PCR products that were amplified from *N. spinipes* DNA extracts, one clone library for each treatment and control, 30 clones per library. Cloning was performed by using the QIAGEN PCR Cloning Kit (Hilden, Germany) according to manufacturer's recommendations. Plasmid DNA was isolated by using the QIAGEN Miniprep Purification System according to manufacturer's recommendations. Sequencing of inserted 16S rRNA gene fragments were performed by using the fD1 primer on a 96-capillary ABI3730XL DNA Analyzer (Applied Biosystems) by Uppsala Genome Center (Uppsala, Sweden). 16S rRNA genes were compared to sequences in the GenBank database by using the blastn software [27]. The obtained sequences were deposited in GenBank (accession numbers FR871410–FR871418 and FR878012–FR878031). Sequences sharing ≥97% base-pair similarity were considered a single phylotype and named according to their closest blastn match [28], [29]. Sequences representing uncultured Bacteroidetes sp. and *Cardinium* shared <97% identity with their closest matching blastn hit, and they were named according to the closest match at division and genus levels, respectively.

### Identification of *Cardinium* bacteria using PCR and specific primers

When using degenerate PCR primers, 16S rRNA gene sequences representing *Cardinium* were only detected in clone libraries created from antibiotic treatments. Hence, to confirm presence of *Cardinium* in the controls, specific probing was carried using the forward 27F-primer, targeting a broad group of bacterial 16S rDNA [25], and the *Cardinium*-specific reverse primer (5′-GTGGATCACTTAACGCTTTCG-3′), with reaction conditions optimized for these primers [8]. The resulting PCR products were sequenced on an ABI 3730 and 3130XL PRISM® DNA Analyzer (KIGene sequencing facility, Solna, Sweden) using both the forward and the reverse primers and the sequence was deposited in GenBank (Accession number: JQ364959).

### Data analysis and statistics

Multiple sequence alignments were performed using the MUSCLE software, version 3.5 [30]. Phylogenetic trees were constructed by using the ML method [31] with the software PHYML version 2.4 [32] and visualized with the program MEGA 3.1 [33]. The ML tree representing all sequenced 16S rRNA genes in this study was unrooted while the ML tree representing *Cardinium* bacteria group A. B, C and D was rooted with a closely related bacterial symbiont in *Amoebophilicus asiaticu*s [34].

The Brillouin diversity index (*H_B_*) and Brillouin relative evenness (*V*) [35] were calculated using number of clones for each phylotype from each clone library:
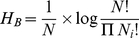
(1)and
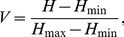
(2)where *N_i_* is the number of clones in the *i* ^th^ phylotype and *N* is the total number of clones, *H*_min_ and *H*_max_ are Brillouin minimum and maximum diversities, respectively:
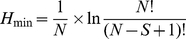
(3)and
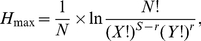
(4)where *S* is the total number of phylotypes, *X* is the integer portion of *N/S*, *Y* is *X*+1 and *r* is the remainder of *X*. The Brillouin indices are not influenced by phylotype richness and are the most appropriate for these data as the selectivity of the PCR approach implies that the sample may not be random [36]. Moreover, a low number of unique phylotypes found in each clone library indicates that these libraries approach censored communities that these indices are suited for [35]. To compare diversity indices between all antibiotic-treated and all control bacterial communities, an unpaired t-test was applied.

As a measure of copepod development, two indices were applied, percentage of individuals that reached a copepodite stage (%Copepodites) and the development index (DI); both were calculated using abundance of nauplii and copepodites at day 13 for ciprofloxacin and sulfamethoxazole treatments and day 11 for the trimethoprim treatment. The DI incorporates survival and metamorphosis success in copepods [37] and was calculated as:
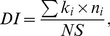
(5)where *k_i_* is assigned stage value (1 for nauplii and 2 for copepodites), *n_i_* number of copepods at that stage, and *NS* – total number of individuals staged.

To evaluate treatment effects on survivorship, %Copepodites and DI, an unpaired t-test was applied for each antibiotics and its respective control system, whereas between-control comparisons were done using a one-way ANOVA. For these tests the data were Box Cox transformed to stabilize distributions. To correlate indices of copepod development and bacterial diversity, a Spearman rank correlation was applied on the non-transformed data.

## Results

### Effects of antibiotics on copepod development

**Figure 1:**
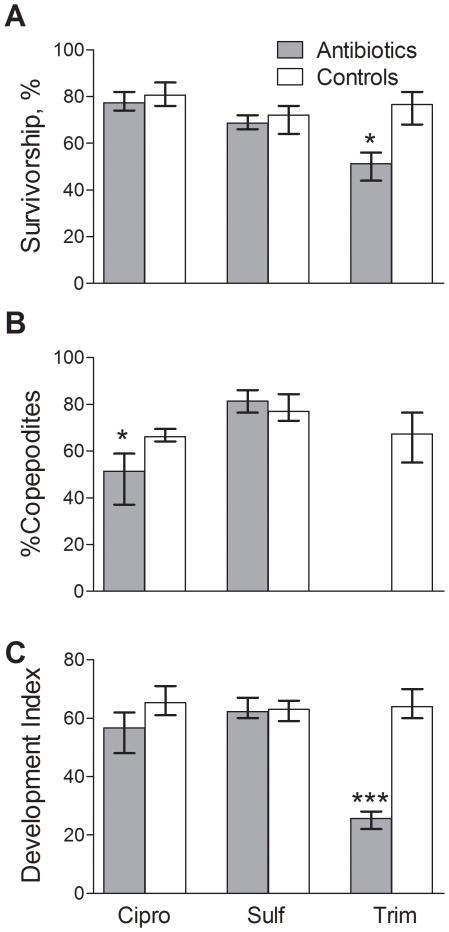
Survival and development of antibiotic-treated and control groups of *Nitocra spinipes*. (A) Survivorship, %; (B) percentage of copepodites (%Copepodites); and (C) copepod developmental index (DI). All data are shown as median and range observed at the termination of experiments, i.e., day 13 for ciprofloxacin (Cipro) and sulfamethoxazole (Sulf), and day 11 for trimethoprim (Trim) treatments, whereas statistical comparisons are based on the Box Cox transformed data; * (p<0.05) and *** (p<0.001) denote significant differences from the respective controls.

A significantly lower survivorship was observed in the trimethoprim treatment compared to the respective control (unpaired t-test, t_4_ = 3.46, *p*<0.026; Figure 1A), whereas no other antibiotic treatments deviated significantly from their respective controls (*p*>0.05 in both cases). In all controls and in ciprofloxacin and sulfomethoxazole treatments, 37–86% of the nauplii had developed into copepodites, whereas the development was arrested in the trimethoprim treatment, where no copepodites were observed (Figure 1B). Moreover, a significantly lower proportion of copepodites occurred in ciprofloxacin-treatment compared to its control (t_4_ = 3.09, *p*<0.037). This, however, was not true for the animals treated with sulfamethoxazole, where no difference from the respective control was observed (t_4_ = 0.48, *p*>0.42). In the ciprofloxacin and sulfomethoxazole treatments, the DI values were not significantly different from the respective controls (*p*>0.17 in both cases; Figure 1C), whereas in trimethoprim treatment, DI was significantly lower than in the control (t_4_ = 3.96, *p*<0.016). There were also significant differences between the solvent controls (F_2,6_ = 15.2, *p*<0.005), with the acetone control having a higher percentage of copepodites than controls with synthetic sea water and with DMSO (Tukey HSD test; *p*<0.048 and *p*<0.004, respectively).

### Bacterial diversity

**Figure 2:**
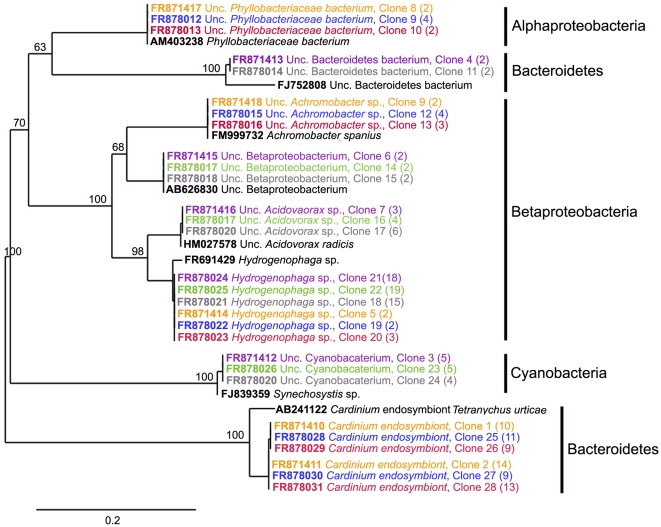
Unrooted 16S rRNA gene tree based on maximum likelihood analyses of bacteria associated with *Nitocra spinipes*. Treatments: ciprofloxacin (orange), sulfomethoxazole (blue), trimethoprim (red); Controls: SS (grey), acetone (purple) and DMSO (green). Reference sequences are in black. Sequences are presented with their GenBank accession numbers followed by identity descriptions. Number of 16S rRNA gene sequences identified in each clone library is indicated in brackets following the clone number. Identical sequences found within a clone library were not included in the tree. One thousand bootstrapped replicate resampled datasets were analyzed. Bootstrap values are indicated as percentage and not shown if below 50%.

The combined library of 180 clones contained 8 phylotypes, with 5 and 4 phylotypes identified in the controls and the antibiotic treatments, respectively (Table 1). Only one phylotype, representing an uncultured *Hydrogenophaga* sp., was shared between the controls and antibiotic treatments. In controls, this phylotype contributed with 50–60% to the total 16S rRNA gene sequences, while its contribution decreased to 7–10% in the antibiotic treatments (Table 1). Altogether, phylotypes representing the Betaproteobacteria division (i.e., uncultured strains of *Hydrogenophaga* sp., *Acidovorax* sp. and a Betaproteobacterium) were the most dominant in controls, contributing with 77–87% to the total 16S rRNA gene sequences (Table 1, Figure 2). By contrast, in the clone libraries from the antibiotic treatments, the Betaproteobacteria (uncultured *Hydrogenophaga* sp. and *Achromobacte*r sp.) were much less represented (13–20%; Table 1). In addition, two other phylotypes representing an uncultured *Cyanobacterium* sp. and an uncultured *Bacteroidetes* sp. were present in the controls (7–17%; Table 1), but not in the antibiotic treatments.

**Table 1:** Relative diversity of bacterial 16S rRNA gene clones associated with *Nitocra spinipes* in antibiotics and control treatments.

Organism	Frequency, %, and number of clones in each library					
(closest match NCBI, its % identity and GenBank Accession No)	Antibiotic treatments			Controls		
	*Cipr*	*Sulf*	*Trim*	*SS*	*Ac*	*DMSO*
1. Unc. *Hydrogenophaga* sp.	7	7	10	50	60	63
(99%, FR691429)	(2)	(2)	(3)	(15)	(18)	(19)
2. Unc. *Acidovorax* sp.	nd	nd	nd	20	10	13
(99%, HM027578)				(6)	(3)	(4)
3. Unc. *Betaproteobacterium* sp.	nd	nd	nd	7	7	7
(99%, AB626830)				(2)	(2)	(2)
4. Unc. Bacteroidetes bacterium	nd	nd	nd	10	7	nd
(92%, FJ752808)				(3)	(2)	
5. Unc. *Cyanobacterium* sp.	nd	nd	nd	13	17	17
(99%, FJ 839359)				(14)	(5)	(5)
6. *Cardinium* endosymbiont	80	67	73	nd	nd	nd
(93–94%, AB506778)	(24)	(20)	(22)			
7. Unc. *Phyllobacteriaceae* sp.	7	13	7	nd	nd	nd
(99%, AM403238)	(2)	(14)	(2)			
8. Unc. *Achromobacter* sp.	7	13	10	nd	nd	nd
(99%, FM626830)	(2)	(14)	(3)			

Unc., Uncultured; nd, 16S rRNA sequence not detected; Treatments: ciprofloxacin (Cipr), Sulfomethoxazole (Sulf), trimethoprim (Trim); Controls: synthetic seawater (SS), acetone (Ac), dimethylsulfoxid (DMSO). Percentages are rounded to the nearest whole number and the corresponding number of clones per library is given in brackets.

The most intriguing finding was, however, identification of bacteria closely related to the genus *Cardinium*. Moreover, these bacteria were most prevalent in the clone libraries from all antibiotic treatments, contributing with 67–87% of the sequenced 16S rRNA genes, whereas none were found in the control libraries (Table 1, Figure 2). However, when *Cardinium*-specific PCR primers were used, the target 16S rRNA gene sequences were identified in all clone libraries, both from controls and the antibiotic-treated copepods. Other phylotypes present in the antibiotic treatments where they contributed with 7–10% each were an uncultured *Phyllobacteriaceae* sp. (Alphaproteobacteria) and an uncultured *Achromobacter* sp. (Betaproteobacteria).

### Differences in bacterial diversity and correlations with the copepod development

Differences in diversity and evenness indices between the controls and antibiotic treatments were significant (Table 2), with the control communities having higher *H_B_* (unpaired t-test; t_4_ = 2.82, *p* = 0.048) and *V* (t_4_ = 2.79, *p* = 0.049) values. There were significant positive correlations between copepod *DI* and diversity indices (Spearman rank r = 0.89 and 0.88 for *H_B_* and *V*, respectively; *p*<0.05 in both cases; *n* = 6). These correlations remained high even when the most deviating trimethoprim treatment was omitted from the data set (Spearman rank r = 0.9 for both *H_B_* and *V*; *n* = 5).The correlations between either survivorship or %Copepodites and any of the diversity indices were not significant (*p*>0.05 in all cases), due to the highly deviating %Copepodites and survivorship values in the trimethoprim treatment.

**Table 2:** Bacterial diversity indices (*H_B_* and *V*) based on clone library analysis.

Treatments	Cipr	Sulf	Trim	SS	Ac	DMSO
***H_B_***	0.59	0.84	0.73	1.15	1.01	0.89
***V***	0.26	0.51	0.40	0.74	0.60	0.57

Treatments: ciprofloxacin (Cipr); Sulfomethoxazole (Sulf); trimethoprim (Trim); Controls: synthetic seawater (SS), acetone (Ac), dimethylsulfoxid (DMSO).

### Phylogenetic analysis of *Cardinium* 16S rRNA genes

**Figure 3:**
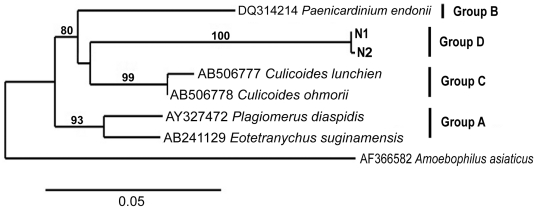
Rooted 16S rRNA gene tree based on maximum likelihood analysis of *Cardinium* sequences. Sequences representing groups A, B, C [43] and the novel group D (this study) are included. The tree was rooted with a 16S rRNA gene sequence associated with an endosymbiont of *Acantamoeba* sp. (Bacteroidetes). One thousand bootstrapped replicate re-sampled datasets were analyzed; bootstrap values are indicated as percentage.

Two ribotypes that shared 99% sequence identity and were most closely related to *Cardinium* (93 and 94% sequence identity, Table 1) were identified in juvenile *N. spinipes*. Two nucleotide transitions at positions 42 bp and 254 bp were responsible for the differences between these ribotypes. To elucidate the phylogenetic relationships between these two highly similar 16S rRNA sequences and known *Cardinium* 16S rRNA groups A, B and C that occur in other arthropods [34], phylogenetic analyses were performed (Figure 3). In the ML tree, the two novel 16S rRNA sequences form a monophyletic group (70% bootstrap support) and clade most closely with group C representing *Cardinium* associated with the biting midge genus *Culicoides* (Figure 3). The ML tree suggests that our 16S rRNA sequences comprise a novel, phylogenetically coherent *Cardinium* group, for which we propose the name “group D”. Sequence alignment (not shown here), suggests that groups C and D are the closest sister clades, sharing 93–94% of sequence identity. Thus, all *Cardinium*-like bacteria known to date can be divided into four groups, with groups A, B and C associated with terrestrial arthropods and group D present in the harpacticoid copepod *N. spinipis*.

## Discussion

As hypothesized, the exposure of *Nitocra spinipes* nauplii to antibiotics altered the taxonomic composition of copepod-associated bacterial communities and reduced their diversity and evenness. Moreover, development in ciprofloxacin and trimethoprim treated copepods was significantly retarded, with a complete arrest in trimethoprim, coinciding with significantly poorer survivorship. Although, the developmental alterations were not apparent in the sulfomethoxazole treatment and varied significantly among the controls, the overall positive correlations between different measures of bacterial diversity and copepod development were significant. Based on these findings, we suggest that compromised juvenile development was, at least partially, related to changes in structure and, possibly, abundance of bacterial communities living in symbiosis with *N. spinipes*. Thus, bacteria-mediated traits in copepods may be altered by antibiotics, a recently recognized type of environmental contaminants, which can result in both short- and long-term consequences for the host fitness.

The retarded development in nauplii exposed to ciprofloxacin and trimethoprim and poor survival in the trimethoprim treatment may have resulted from (1) direct antibiotic toxicity, (2) decreased food intake, (3) an indirect effect on the symbiotic bacteria involved in basic physiological functions, such as food digestion, and (4) infections resulted from an outburst of a virus or an antibiotic-resistant pathogenic microorganism. None of these potential mechanisms are mutually exclusive, and, unfortunately, our experimental design does not allow unambiguous determination of their relative contributions to the observed responses.

The first mechanism is, however, not likely to contribute substantially, since the antibiotic concentrations that we used (3–8 mg/L) were well below the acute toxicity values for another microcrustacean, the cladoceran *Daphnia magna*: 48 h EC_50_; ciprofloxacin: >60 mg/L, trimethoprim: 123 mg/L [22], and sulfamethoxazole: >100 mg/L [38], [39]. It should be noted, however, that similar to our study, these values do not represent the direct toxicity levels, but rather a combined response of the host and its symbiotic bacterial community. The concentrations applied in our study were also below the antibiotic levels used in eukaryotic cell cultures to avoid microbiological contamination [40]. To delineate direct and bacteria-mediated effects, *in vitro* tests using relevant cell cultures would be the most helpful for the identification of cytotoxic effects and/or *in vivo* tests with antibiotics specifically targeting bacterial cells and exhibiting particularly low toxicity to eukaryotes, e. g., β-lactams [40].

The decreased food intake is also not likely to be the main mechanism behind the observed decrease in the copepod development success, although it may be a contributing factor. Indeed, given the generally low toxicity of antimicrobial substances for crustaceans, it has been suggested that if antibiotics exert adverse effects on these organisms in nature, these effects could be an indirect result of suppressing their prey [39]. In copepod cultures, bacteria are normally present in the media, where they grow using dissolved material from copepod sloppy feeding, excretion and leakage from fecal pellets [16], [17]. Like other omnivorous harpacticoids, *N. spinipes* may satisfy their nutrition requirements by feeding on bacteria, particularly during naupliar development [41]. In controls, free-living bacteria were likely to be more abundant and diverse compared to the antibiotic treatments, and hence their contribution to the copepod diet would be higher. However, algae are of superior food quality for benthic harpacticoids [41], [42], and, together with the negligibly low bacterial biomass relative to the algae that were at surplus, this suggests that bacteria were not a quantitatively important prey in the experiment. Nevertheless, these antibiotic-driven changes in the surrounding bacterial assemblages may have important implications for the gut microflora, as copepod feeding brings new bacteria to its gut [16].

The compromised development and survival in the antibiotic treatments is likely to be associated with the disruption of mutualistic interactions between symbiotic bacteria and their copepod hosts as indicated by significant correlations between copepod development index and bacterial diversity indices. In insects, for example, antibiotics selectively eliminated physiologically important gut microbiota, leading to lower reproduction and fitness [43]. Since in crustaceans bacteria-mediated digestion contributes greatly to breakdown and absorption of many essential compounds, such as essential amino acids and vitamins [44], the growth penalty in copepods with compromised microflora is to be expected. The main evidence for the primary effects on bacteria rather than on the copepods is that the composition of clone libraries was similar among the antibiotic treatments (Table 1), suggesting strong effects on the bacterial composition and relative frequencies of different taxa. This is in line with previous ecotoxicological studies, where observable effect concentrations of antibiotics on bacteria and microalgae were 2 to 3 orders of magnitude below those for higher trophic levels, such as crustaceans and fish. However, it is also possible that similarities of clone libraries within controls and antibiotic treatments were due to the fact that these libraries were generated by using a single pair of primers that may only cover a fraction of the true diversity [45]. It is also important to point out that absolute abundance of different bacterial ribotypes was not measured in our study, whereas variation not only in community composition, but also bacterial abundance, may explain differential responses of the copepods exposed to different antibiotics.

Finally, viral or antibiotic-resistant bacterial infections may have contributed to the observed mortalities, particularly in the trimethoprim treatment, similar to the penicillin-induced activation of a latent virus in rodents [46]. In guinea pigs, the rapid alteration of intestinal flora from predominantly Gram-positive bacteria to mostly Gram-negative following the administration of penicillin has been implicated in highly lethal outburst of infectious disease [46]. In invertebrates, as in many other animals, microflora has a continuous and dynamic effect on the host's gut and systemic immune responses. Therefore, antibiotic-induced suppression of bacteria involved, for example, in synthesis of gut mucosa to produce antibodies to pathogens, would result in lowering immune status and higher susceptibility of the animal to bacterial and viral infections [44]. The gut-related immune modulators are also involved in the host's response to endosymbionts and it has been proposed that immune function keeps endosymbionts under control [44], [47]. Alternative evidence is accumulating that vertically transmitted endosymbionts, such as *Cardinium*, may protect their hosts from horizontally transmitted pathogens [47]. Thus, the increased *Cardinium* frequencies in the antibiotic treatments might result from the both weakened immune status and increased pathogen abundance in the system. It is, however, also possible that *Cardinium* abundance did not change but became detectable because the relative contribution of its DNA to the total bacteria DNA in the sample increased when other bacteria were suppressed by the antibiotics. This would result in over-represented *Cardinium* sequences in the clone libraries from the antibiotic treatments.

The bacteria-mediated processes in copepods are largely unexplored, but in crustaceans, symbiotic bacteria are important for nutrition, detoxification of organic pollutants, mediation of disease resistance and immune defense, and other complex interactions that have been established during their co-evolutionary histories [1]. Therefore, to interpret disturbances in bacteria-copepod homeostasis, we need to know what these bacteria are and in which environments they occur. All bacteria identified in this study are taxonomically similar to those previously identified as both free-living and symbiotic in a variety of aquatic and terrestrial environments and hosts. For example, a cyanobacterium identified in controls shared 99% 16S rRNA gene sequence identity with the symbiotic cyanobacterium *Synechocystis* sp. (Table 1), previously isolated from wheat root cells where it contributed to biosynthesis of growth hormones [48]. Other symbiotic cyanobacteria have been identified in the gut microflora of the freshwater copepod *Eudiaptomus gracilis* [18]. Considering relatively high sensitivity of cyanobacteria to antibiotics [49], it is not surprising that *Synechocystis* was absent in the clone libraries of antibiotic-treated animals. Another bacterium, also unique for the control treatments, a *Bacteroidetes* sp., shares 92% 16S rRNA gene sequence identity with a bacterium previously isolated from the burrowing walls of the worm *Nereis diversicolor* (Pischedda et al., unpublished sequence, NCBI). An uncultured *Hydrogenophaga* sp. was identified most frequently in controls, whereas its contribution to the clone libraries in antibiotic treatments decreased 6- to 8-fold (Figure 2; Table 1). Its closest described neighbor in terms of 16S rRNA gene sequence identity was a *Hydrogenophaga* sp. isolated from a lake in Antarctica (Peeters et al., unpublished sequence, NCBI). Other closely related *Hydrogenophaga* species have been reported to be capable of benzene and chlorinated biphenyl degradation [50]. A close relative to another potent chlorinated organic compound-degrader was *Achromobacter* sp. identified in all control treatments. This ribotype shared 99% 16S rRNA gene sequence identity with an *Achromobacter spanius* (unpublished sequence, NCBI) that was previously observed to use pentachlorophenol as the sole carbon source in mixed culture. In antibiotic treatments, we identified a *Phyllobacteriaceae* sp. that shared 99% of 16S rRNA gene sequence identity with a nitrate reducing and denitrifying bacteria isolated from a water treatment system in a marine aquaculture facilities (unpublished sequence, NCBI). Other closely related *Phyllobacteriaceae* spp. were cloned from the guts of various invertebrates: larval Asian longhorned beetle [51], earthworms [52], and ants [53]. Thus, *N. spinipes* harbors phylotypes identical or very closely related to those known as gut microflora in various aquatic and terrestrial invertebrates, albeit the functional relationships between specific bacteria and their hosts are rarely understood. In this study, we have shown that bacterial communities changed dramatically at low concentrations of antibiotics and that higher bacterial diversity was associated with successful development and survival. To understand the mechanisms of this linkage, it is essential to investigate specific functional relationships between the copepods and their associated bacterial flora, as well as localization and abundances of various bacteria by using, for example, Next Gen sequencing, advanced microscopy techniques coupled with gene probing [54] and isotopic labeling [53].

The discovery of *Cardinium* bacteria associated with *N. spinipes* is intriguing as this intracellular endosymbiont has been implicated in a variety of different reproductive manipulations: feminizing and/or forcing asexuality on its host, incompatibility between infected males and uninfected (or differently infected) females, and killing males [10]–[12]. It has been reported to cause these disorders in terrestrial arthropods, such as parasitoid wasps, spider mites, planthoppers and oleander scales [8], [10]–[12], [55], but to the best of our knowledge, *Cardinium* has never been reported to occur in crustaceans. Based on a recent taxonomic revision [34], bacteria within the genus *Cardinium* fall into three phylogenetically distinct sister clades (groups A, B and C). Group A, previously referred to as “Candidatus *Cardinium hertigii*”, is present in various arthropod species; group B, previously referred to as “Candidatus *Paenicardinium endonii*”, is present in plant parasitic nematodes; and group C is present in biting midges, *Culicoides*. On the basis of morphological and molecular data, these groups were integrated as Candidatus *Cardinium hertigii* species referred to as *Cardinium* [34]. Using phylogentic analysis of the two highly similar *Cardinium*-ribotypes identified in our study and other *Cardinium* previously described in arthropods and nematodes, we identified a new *Cardinium* group, for which we propose the name “group D” (Figs. 2 and 3). Interestingly, this clade is most closely to the *Cardinium* isolated from *Culicoides*, a genus whose larval stages develop in water, such as coastal areas and shallow ponds. It remains to be seen how widespread *Cardinium* is among aquatic crustaceans in general and copepods in particular, and what are the phylogenetic relationships in various *Cardinium*-host systems. Of particular interest, of course, is the understanding of the very nature of *Cardinium*-copepod symbiosis, the role of the bacterium in host reproduction and its ecological implications.

Female-biased adult sex ratios are common, both in wild populations and in cultures of various copepod species [56], including harpacticoids [57]. This bias is important, because density of males to fertilize females might be a limiting factor to population growth [56]. Most commonly, biased sex ratios are explained by sex- or stage specific differences in mortality [58], but there are very few experimental evidence for that in general and none for the early life stages. Interestingly, endosymbiotic bacteria affecting sex determination have been hypothesized to occur in harpacticoid copepods to explain variations in sex ratio; however all attempts to detect their presence failed [59]. Recently, another explanation was advocated: environmentally controlled sex change is an important mechanism determining the adult sex ratio [56]. Based on the discovery of *Cardinium* in *N. spinipes*, we suggest that, at least in this species, biased sex ratios, resulting – among other reasons – from sex change and differential mortality during ontogeny, might be related to infestation with *Cardinium*. Unfortunately, the design of our experiment did not allow us to determine sex ratio, because sex cannot be morphologically distinguished until the fourth-fifth copepodite stage, whereas our test animals were younger upon the termination of the experiment. Measuring abundances of *Cardinium* in specific ontogenetic stages on individual basis by a quantitative real-time PCR [60] would complement clone library approach when studying the infection in relation to reproduction and population sex ratio in various ecological settings.

Through the use and release of antimicrobial substances to the environment, lasting alterations are being made to a mutualistic relationship that has taken millennia to evolve: the relationship between the host and its microbiota. Our study has demonstrated that antibiotics caused substantial changes in copepod-associated bacterial communities, and these changes have a potential to affect long-term fitness and survival of the copepods. At the same time, different groups of antibiotics may be used to manipulate bacterial diversity and abundance in test organisms, thus providing the possibility to study functional roles of specific bacteria in host physiology. Indeed, probing with various antibiotics can be applied to yield information regarding various bacterial functions and complexity of bacterial networks within a host [61]. Testing different antibiotics for their potency to affect non-target species by disrupting their symbiotic relationships may contribute greatly to both predicting environmental effects of pharmaceutical contamination and understanding microbial-host interactions.

## Supporting Information

Data S1 **Method description for chemical analysis used to measure actual concentrations of the test substances (ciprofloxacin, sulfomethoxazole and trimethoprim).**
(DOC)

## References

[1] Toft C, Andersson SGE (2010). Evolutionary microbial genomics: Insights into bacterial host adaptation.. Nat Rev Genet.

[2] Sachs JL, Essenberg CJ, Turcotte MM (2011). New paradigms for the evolution of beneficial infections.. Trends Ecol Evol.

[3] Werren JH (1997). Biology of Wolbachia.. Annu Rev Entomol.

[4] Douglas AE (1998). Nutritional interactions in insect-microbial symbioses: aphids and their symbiotic bacteria *Buchnera*.. Annu Rev Entomol.

[5] Wilson EO (1992). The diversity of life.

[6] Kurtti TJ, Munderloh UG, Andreadis TG, Magnarelli LA, Mather TN (1996). Tick cell culture isolation of an intracellular prokaryote from the tick *Ixodes scapularis*.. J Invert Pathol.

[7] Zchori-Fein E, Perlman SJ, Kelly SE, Katzir N, Hunter MS (2004). Characterization of a ‘Bacteroidetes’ symbiont in *Encarsia wasps* (Hymenoptera: Aphelinidae): proposal of ‘Candidatus *Cardinium hertigii*’.. Int J Syst Evol Microbiol.

[8] Zchori-Fein E, Perlman SJ (2004). Distribution of the bacterial symbiont *Cardinium* in arthropods.. Mol Ecol.

[9] Hilgenboecker K, Hammerstein P, Schlattmann P, Telschow A, Werren JH (2008). How many species are infected with *Wolbachia*? – A statistical analysis of current data.. FEMS Microbiol Lett.

[10] Hunter MS, Perlman SJ, Kelly SE (2003). A bacterial symbiont in the Bacteroidetes induces cytoplasmic incompatibility in the parasitoid wasp *Encarsia pergandiella*.. Proc R Soc Lond B Biol Sci.

[11] Gotoh T, Noda H, Ito S (2007). *Cardinium* symbionts cause cytoplasmic incompatibility in spider mites.. Heredity.

[12] Ros VI, Breeuwer JA (2009). The effects of, and interactions between, *Cardinium* and *Wolbachia* in the doubly infected spider mite *Bryobia sarothamni*.. Heredity.

[13] Parsons TR, Takahashi M, Hargrave B (1977). Biological Oceanographic Processes..

[14] Støttrup J (2000). The elusive copepods: their production and suitability in marine aquaculture.. Aquacult Res.

[15] Raisuddin S, Kwok KWH, Leung KMY, Schlenk D, Lee JS (2007). The copepod *Tigriopus*: A promising marine model organism for ecotoxicology and environmental genomics.. Aquat Toxicol.

[16] Tang K, Dziallas C, Hutalle-Schmelzer K, Grossart HP (2009). Effects of food on bacterial community composition associated with the copepod *Acartia tonsa* Dana.. Biology Letters.

[17] Tang KW, Turk V, Grossart HP (2010). Linkage between crustacean zooplankton and aquatic bacteria.. Aquat Microb Ecol.

[18] Homonnay ZG, Kéki Z, Márialigeti K, Tóth EM (2011). Bacterial communities in the gut of the freshwater copepod *Eudiaptomus gracilis*.. J Basic Microbiol.

[19] Kümmerer K (2009). Antibiotics in the aquatic environment – A review – Part I.. Chemosphere.

[20] Grung M, Källqvist T, Sakshaug S, Skurtveit S, Thomas KV (2008). Environmental assessment of Norwegian priority pharmaceuticals based on the EMEA guideline.. Ecotoxicol Environ Safety.

[21] Grung M, Källqvist T, Sakshaug S, Skurtveit S, Thomas KV (2008). Environmental assessment of Norwegian priority pharmaceuticals based on the EMEA guideline.. Ecotoxicol Environ Safety.

[22] Halling-Sorensen B, Holten Lutzhoft HC, Andersen HR, Ingerslev F (2000). Environmental risk assessment of antibiotics; comparison of mecillinam, trimethoprim and ciprofloxacin.. J Antimicrob Therapy.

[23] Robinson AA, lden JB, Lydy MJ (2005). Toxicity of fluoroquinolone antibiotics to aquatic organisms.. Environ Toxicol Chem.

[24] Straughan DJ, Lehman N (2000). Genetic differentiation among Oregon lake populations of the *Daphnia pulex* species complex.. J Heredity.

[25] Weisburg WG, Barns SM, Pelletier DA, Lane DJ (1991). 16S ribosomal DNA amplification for phylogenetic study.. J Bacteriol.

[26] Muyzer G, Teske A, Wirsen CO, Jannasch HW (1995). Phylogenetic relationships of Thiomicrospira species and their identification in deep-sea hydrothermal vent samples by denaturing gradient gel electrophoresis of 16S rDNA fragments.. Arch Microbiol.

[27] Altschul SF, Madden TL, Schffer AA, Zhang J, Zhang Z (1997). Gapped BLAST and PSI-BLAST: a new generation of protein database search programs.. Nucl Acids Res.

[28] Stackebrandt E, Goebel BM (1994). Taxonomic note: a place for DNA-DNA reassociation and 16s rRNA sequence analysis in the present species definition in bacteriology.. Int J Syst Bacteriol.

[29] Hagström A, Pommier T, Rohwer F, Simu K, Stolte W (2002). Use of 16S ribosomal DNA for delineation of marine bacterioplankton species.. Appl Environ Microbiol.

[30] Edgar RC (2004). MUSCLE: a multiple sequence alignment method with reduced time and space complexity.. BMC Bioinformatics.

[31] Felsenstein J (1981). Evolutionary trees from DNA sequences: a maximum likelihood approach.. J Mol Evol.

[32] Guindon S, Lethiec F, Duroux P, Gascuel O (2005). PHYML Online—a web server for fast maximum likelihood-based phylogenetic inference.. Nucl Acids Res.

[33] Kumar S, Tamura K, Nei M (2004). MEGA3: integrated software for molecular evolutionary genetics analysis and sequence alignment.. Briefings in Bioinformatics.

[34] Nakamura Y, Kawai S, Yukuhiro F, Ito S, Gotoh T (2009). Prevalence of Cardinium bacteria in planthoppers and spider mites and taxonomic revision of “Candidatus *Cardinium hertigii*” based on detection of a new *Cardinium* group from biting midges.. Appl Environ Microbiol.

[35] Magurran AE (1988). Ecological diversity and its measurement.

[36] Wilberforce EM, Boddy L, Griffiths R, Griffith GW (2003). Agricultural management affects communities of culturable root-endophytic fungi in temperate grasslands.. Soil Biol Biochem.

[37] Knuckey RM, Semmens GL, Mayer RJ, Rimmer MA (2005). Development of an optimal microalgal diet for the culture of the calanoid copepod *Acartia sinjiensis*: Effect of algal species and feed concentration on copepod development.. Aquaculture.

[38] Ferrari B, Mons R, Vollat B, Fraysse B, Paxéus N (2004). ERA of six human pharmaceuticals: are the current ERA procedures sufficient for the protection of the aquatic environment?. Environ Toxicol Chem.

[39] Wollenberger L, Halling-Sørensen B, Kusk KO (2000). Acute and chronic toxicity of veterinary antibiotics to *Daphnia magna*.. Chemosphere.

[40] Kuhlmann I (1996). The prophylactic use of antibiotics in cell culture.. Cytotechnology.

[41] Rieper M (1982). Feeding preferences of marine harpacticoid copepods for various species of bacteria.. Mar Ecol Prog Ser.

[42] Ustach JF (1982). Algae, bacteria and detritus as food for the harpacticoid copepod, *Heteropsyllus pseudonunni* Coull and Palmer.. J Exp Mar Biol Ecol.

[43] Giordano R, Weber E, Waite J, Bencivenga N, Krogh PH (2010). Effect of a high dose of three antibiotics on the reproduction of a parthenogenetic strain of *Folsomia candida* (Isotomidae: Collembola).. Environ Entomol.

[44] Harris JM (1993). The presence, nature, and role of gut microflora in aquatic invertebrates: A synthesis.. Microb Ecol.

[45] Jeon S, Bunge J, Leslin C, Stoeck T, Hong S (2008). Environmental rRNA inventories miss over half of protistan diversity.. BMC Microbiol.

[46] Green RH (1974). The association of viral activation with penicillin toxicity in Guinea pigs and hamsters.. Yale J Biol Medicine.

[47] Haine ER (2008). Symbiont-mediated protection.. Proc R Soc Lond B.

[48] Ahmed M, Stal LJ, Hasnain S (2010). Association of non-heterocystous cyanobacteria with crop plants.. Plant Soil.

[49] van der Grinten E, Pikkemaat MG, van den Brandhof EJ, Stroomberg GJ, Kraak MH (2010). Comparing the sensitivity of algal, cyanobacterial and bacterial bioassays to different groups of antibiotics.. Chemosphere.

[50] Lambo AJ, Patel TR (2006). Isolation and characterization of a biphenyl-utilizing psychrotrophic bacterium, *Hydrogenophaga taeniospiralis* IA3-A, that cometabolize dichlorobiphenyls and polychlorinated biphenyl congeners in Aroclor 1221.. J Basic Microbiol.

[51] Geib SM, Jimenez-Gasco Mdel M, Carlson JE, Tien M, Hoover K (2009). Effect of host tree species on cellulase activity and bacterial community composition in the gut of larval Asian longhorned beetle.. Environ Entomol.

[52] Wüst PK, Horn MA, Drake HL (2011). Clostridiaceae and Enterobacteriaceae as active fermenters in earthworm gut content.. ISME J.

[53] Russell JA, Moreaua CS, Goldman-Huertas B, Fujiwara M, Lohman DJ (2009). Bacterial gut symbionts are tightly linked with the evolution of herbivory in ants.. Proc Natl Acad Sci U S A.

[54] Rawls JR, Mahowald MA, Goodman AL, Trent CM, Gordon JI (2007). In vivo imaging and genetic analysis link bacterial motility and symbiosis in the zebrafish gut.. Proc Natl Acad Sci U S A.

[55] Provencher LM, Morse GE, Weeks AR, Normark BB (2005). Parthenogenesis in the *Aspidiotus nerii* Complex (Hemiptera: Diaspididae): A Single Origin of a Worldwide, Polyphagous Lineage Associated with *Cardinium* Bacteria.. Ann Entomol Soc Am.

[56] Gusmão LFM, McKinnon AD (2009). Sex ratios, intersexuality and sex change in copepods.. J Plankton Res.

[57] Thistle D, Eckman JE (1990). What is the sex ratio of harpacticoid copepods in the deep sea?. Mar Biol.

[58] Hirst AG, Bonnet D, Conway DVP, Kiørboe T (2010). Does predation control adult sex ratios and longevities in marine pelagic copepods?. Limnol Oceanogr.

[59] Voordouw MJ, Stebbins G, Robinson HE, Perrot-Minnot MJ, Rigaud T (2008). Genetic variation in the primary sex ratio in populations of the intertidal copepod, *Tigriopus californicus,* is widespread on Vancouver Island.. Evol Ecol Res.

[60] Morimoto S, Kurtti TJ, Noda H (2006). In vitro cultivation and antibiotic susceptibility of a Cytophaga-like intracellular symbiote isolated from the tick *Ixodes scapularis*.. Curr Microbiol.

[61] Falconer SB, Czarny TL, Brown ED (2011). Antibiotics as probes of biological complexity.. Nature Chem Biol.

