# Supplementary material for: Antibiotic-Induced Change of Bacterial Communities Associated with the Copepod Nitocra spinipes
Source: PLoS One. 2012 Mar 12;7(3):e33107. doi: 10.1371/journal.pone.0033107 (PMC3299745; doi:10.1371/journal.pone.0033107)
Supplement: Data S1 — Method description for chemical analysis used to measure actual concentrations of the test substances (ciprofloxacin, sulfomethoxazole and trimethoprim). (DOC) [file pone.0033107.s001.doc]

**Supporting Information**

Edlund et al. Antibiotic-induced Change of Bacterial Communities Associated with the Copepod *Nitocra spinipes*, PLoS ONE, 2012.

**Supplementary Data S1.** *Chemical analysis of antibiotics*

Water samples were analyzed by direct injection on a liquid chromatography- tandem mass-spectrometer. All pharmaceutical reference standards were classified as HPLC grade (>98%). Methanol (hypergrade for LC/MS) and acetonitrile (hypergrade for LC/MS) were purchased from Merck (Darmstadt, Germany). Formic acid (eluent additive for LC-MS >98%) and sulphuric acid were purchased from Sigma-Aldrich (Steinheim, Germany). The purified water was prepared by an Milli-Q Gradient ultra-pure water system (Millipore, Billerica, USA), equipped with a UV radiation source. ^13^C_3_^15^N-ciprofloxacin, ^13^C_6_-sulfamethoxazole and ^13^C_3_-trimethoprim were obtained from Cambridge Isotope Laboratories (Andover, MA, USA).

Water samples were filtered through a 0.45 µm membrane filter (MF, Millipore, Sundbyberg, Sweden), 50 ng of each internal standard were added to each sample and then 20 µm of the samples were injected directly. A triple stage quadrupole MS/MS TSQ Quantum Ultra EMR (Thermo Fisher Scientific, San Jose, CA, USA) coupled with an Accela LC pump (Thermo Fisher Scientific, San Jose, CA, USA) and a PAL HTC autosampler (CTC Analytics AG, Zwingen, Switzerland) were used as analytical system. Samples were loaded onto a Hypersil GOLD aQ TM column (50 mm x 2.1 mm ID x 5 µm particles, Thermo Fisher Scientific, San Jose, CA, USA) preceded by a guard column (2 mm×2.1 mm i.d, 5 µm particles) of the same packing material and from the same manufacturer. A gradient of flow and methanol and acetonitrile in water (all solvents buffered by 0.1% formic acid) was used for elution of analytes. The elution conditions were programmed as follows: 200 µL min^-1^ water for 1 min isocratically, then composition is changed to 30/10/60 water/acetonitrile/methanol and flow of 250 µL min^-1^ at 8 min. Then the column was washed by mixture ACN/ methanol 60/40 and flow of 300 µl min^-1^ in 9 minutes. These parameters were kept for 1 min and then they were switched to starting conditions and equilibrated for 4 min before next run.

Heated electrospray (HESI) in positive mode was used for ionisation of target compounds. Both first and third quadrupole were operated at resolution 0.7 FMWH and two SRM transitions were monitored for each analyte. Samples were quantified using internal standard method. Several calibration standards covering all concentration range were measured before, in the middle and at the end of sample sequences. The maximum difference between results at quantification and qualification mass transition was set to 30% as criterion for positive identification.
